# A Compend of the Diseases of Eye and Refraction

**Published:** 1900-06

**Authors:** 


A Compend of the Diseases of Eye and Refraction, including Surgery. By George M. Gould, A. M., M. D., and Walter L. Pyle, A. M., M. D., Assistant Surgeon to Wills' Eye Hosptial, Philadelphia. Second edition, revised and enlarged. One hundred and nine illustrations, several of which are in colors. Pages, 295; price, 80 cents. Philadelphia: P. Blakiston's Son & Co., 1012 Walnut St. 1899.

This little book has ophthalmology so condensed that a student who needs a hasty review of the subject can obtain it by scanning two hundred and seventy-eight pages. It gives a succinct but comprehensive outline of elementary optics, and teaches how to make a diagnosis by inspection, tension, the ophthalmoscope, the retinoscope, test-cards, test-lenses, mydriatics, cycloplegics, etc. Part Second is devoted to the diseases of the eye, local ocular therapeutics, abbreviations, a glossary and an index.

